# Supplementary material for: Pyruvate Dehydrogenase Kinase 1 inhibition mediated oxidative phosphorylation enhancement in cartilage promotes osteoarthritis progression
Source: BMC Musculoskelet Disord. 2023 Jul 20;24:597. doi: 10.1186/s12891-023-06585-6 (PMC10357736; doi:10.1186/s12891-023-06585-6)
Supplement: Supplementary file 1 — Additional file 1. [file 12891_2023_6585_MOESM1_ESM.zip › BP.Gsea.1653623667859/GOBP_MISMATCH_REPAIR.html]

Details for gene set GOBP\_MISMATCH\_REPAIR[GSEA]

|  || Dataset | 55457\_collapsed\_to\_symbols.cls.cls #OA\_versus\_normal.cls.cls #OA\_versus\_normal\_repos |
| Phenotype | cls.cls#OA\_versus\_normal\_repos |
| Upregulated in class | OA |
| GeneSet | GOBP\_MISMATCH\_REPAIR |
| Enrichment Score (ES) | 0.5120351 |
| Normalized Enrichment Score (NES) | 1.8375853 |
| Nominal p-value | 0.0 |
| FDR q-value | 0.06623582 |
| FWER p-Value | 0.935 |
Table: GSEA Results Summary

  

Fig 1: Enrichment plot: GOBP\_MISMATCH\_REPAIR      
 Profile of the Running ES Score & Positions of GeneSet Members on the Rank Ordered List

  

| SYMBOL | TITLE | RANK IN GENE LIST | RANK METRIC SCORE | RUNNING ES | CORE ENRICHMENT || 1 | RPA3 | replication protein A3 [Source:HGNC Symbol;Acc:HGNC:10291] | 13 | 1.159 | 0.1141 | Yes |
| 2 | RPA2 | replication protein A2 [Source:HGNC Symbol;Acc:HGNC:10290] | 339 | 0.727 | 0.1611 | Yes |
| 3 | TREX1 | three prime repair exonuclease 1 [Source:HGNC Symbol;Acc:HGNC:12269] | 393 | 0.709 | 0.2274 | Yes |
| 4 | MLH1 | mutL homolog 1 [Source:HGNC Symbol;Acc:HGNC:7127] | 497 | 0.660 | 0.2850 | Yes |
| 5 | RPA1 | replication protein A1 [Source:HGNC Symbol;Acc:HGNC:10289] | 571 | 0.632 | 0.3421 | Yes |
| 6 | MSH2 | mutS homolog 2 [Source:HGNC Symbol;Acc:HGNC:7325] | 739 | 0.584 | 0.3872 | Yes |
| 7 | PMS2P1 | "PMS1 homolog 2, mismatch repair system component pseudogene 1 [Source:HGNC Symbol;Acc:HGNC:9123]" | 917 | 0.537 | 0.4268 | Yes |
| 8 | RNASEH2A | ribonuclease H2 subunit A [Source:HGNC Symbol;Acc:HGNC:18518] | 1102 | 0.495 | 0.4617 | Yes |
| 9 | PMS2P3 | "PMS1 homolog 2, mismatch repair system component pseudogene 3 [Source:HGNC Symbol;Acc:HGNC:9128]" | 1412 | 0.442 | 0.4816 | Yes |
| 10 | PCNA | proliferating cell nuclear antigen [Source:HGNC Symbol;Acc:HGNC:8729] | 1557 | 0.419 | 0.5120 | Yes |
| 11 | ERCC1 | "ERCC excision repair 1, endonuclease non-catalytic subunit [Source:HGNC Symbol;Acc:HGNC:3433]" | 2948 | 0.255 | 0.4294 | No |
| 12 | XPC | "XPC complex subunit, DNA damage recognition and repair factor [Source:HGNC Symbol;Acc:HGNC:12816]" | 3278 | 0.222 | 0.4259 | No |
| 13 | MLH3 | mutL homolog 3 [Source:HGNC Symbol;Acc:HGNC:7128] | 4335 | 0.125 | 0.3564 | No |
| 14 | PMS2P5 | "PMS1 homolog 2, mismatch repair system component pseudogene 5 [Source:HGNC Symbol;Acc:HGNC:9130]" | 4623 | 0.103 | 0.3443 | No |
| 15 | TP73 | tumor protein p73 [Source:HGNC Symbol;Acc:HGNC:12003] | 5222 | 0.055 | 0.3032 | No |
| 16 | MUTYH | mutY DNA glycosylase [Source:HGNC Symbol;Acc:HGNC:7527] | 5252 | 0.052 | 0.3061 | No |
| 17 | SETD2 | "SET domain containing 2, histone lysine methyltransferase [Source:HGNC Symbol;Acc:HGNC:18420]" | 6926 | -0.074 | 0.1835 | No |
| 18 | LIG1 | DNA ligase 1 [Source:HGNC Symbol;Acc:HGNC:6598] | 6944 | -0.075 | 0.1896 | No |
| 19 | PMS1 | "PMS1 homolog 1, mismatch repair system component [Source:HGNC Symbol;Acc:HGNC:9121]" | 6968 | -0.077 | 0.1955 | No |
| 20 | POLD3 | "DNA polymerase delta 3, accessory subunit [Source:HGNC Symbol;Acc:HGNC:20932]" | 7568 | -0.125 | 0.1614 | No |
| 21 | EXO1 | exonuclease 1 [Source:HGNC Symbol;Acc:HGNC:3511] | 8088 | -0.162 | 0.1372 | No |
| 22 | PMS2 | "PMS1 homolog 2, mismatch repair system component [Source:HGNC Symbol;Acc:HGNC:9122]" | 8172 | -0.169 | 0.1475 | No |
| 23 | PRKCG | protein kinase C gamma [Source:HGNC Symbol;Acc:HGNC:9402] | 8582 | -0.200 | 0.1356 | No |
| 24 | MCM9 | minichromosome maintenance 9 homologous recombination repair factor [Source:HGNC Symbol;Acc:HGNC:21484] | 8781 | -0.215 | 0.1415 | No |
| 25 | MSH6 | mutS homolog 6 [Source:HGNC Symbol;Acc:HGNC:7329] | 8846 | -0.221 | 0.1585 | No |
| 26 | ABL1 | "ABL proto-oncogene 1, non-receptor tyrosine kinase [Source:HGNC Symbol;Acc:HGNC:76]" | 9081 | -0.238 | 0.1640 | No |
| 27 | MSH3 | mutS homolog 3 [Source:HGNC Symbol;Acc:HGNC:7326] | 9796 | -0.301 | 0.1385 | No |
| 28 | TDG | thymine DNA glycosylase [Source:HGNC Symbol;Acc:HGNC:11700] | 10728 | -0.396 | 0.1055 | No |
| 29 | HMGB1 | high mobility group box 1 [Source:HGNC Symbol;Acc:HGNC:4983] | 12170 | -0.636 | 0.0567 | No |
Table: GSEA details [plain text format]

  

Fig 2: GOBP\_MISMATCH\_REPAIR      
 Blue-Pink O' Gram in the Space of the Analyzed GeneSet

  

Fig 3: GOBP\_MISMATCH\_REPAIR: Random ES distribution      
 Gene set null distribution of ES for **GOBP\_MISMATCH\_REPAIR**
